# HDC1 Promotes Primary Root Elongation by Regulating Auxin and K^+^ Homeostasis in Response to Low-K^+^ Stress

**DOI:** 10.3390/biology14010057

**Published:** 2025-01-12

**Authors:** Xiaofang Kuang, Hao Chen, Jing Xiang, Juan Zeng, Qing Liu, Yi Su, Chao Huang, Ruozhong Wang, Wanhuang Lin, Zhigang Huang

**Affiliations:** Hunan Provincial Key Laboratory of Phytohormones and Growth Development, College of Bioscience and Biotechnology, Hunan Agricultural University, Changsha 410128, China; xiaofangkuang1011@163.com (X.K.); haochen@stu.hunau.edu.cn (H.C.); jingxiang0618@outlook.com (J.X.); 15073151686@163.com (J.Z.); lqing2003@163.com (Q.L.); syui1979@163.com (Y.S.); chaoh@hunau.edu.cn (C.H.); wangruozhong@hunau.edu.cn (R.W.)

**Keywords:** auxin transport, HDC1, low-K^+^ stress, root growth

## Abstract

Plants frequently encounter relatively low and fluctuating potassium (K^+^) concentrations in soil, with roots serving as primary responders to this stress. Histone modifications, such as de-/acetylation, can function as epigenetic markers of stress-inducible genes. However, the signaling network between histone modifications and low-K^+^ (LK) response pathways remains unclear. This study investigated the regulatory role of Histone Deacetylase Complex 1 (HDC1) in primary root growth of *Arabidopsis thaliana* under K^+^ deficiency stress. Using a *hdc1-2* mutant line, we observed that HDC1 positively regulated root growth under LK conditions. Compared to wild-type (WT) plants, the *hdc1-2* mutant exhibited significantly inhibited primary root growth under LK conditions, whereas HDC1-overexpression lines displayed opposite phenotypes. No significant differences were observed under HK conditions. Further analysis revealed that the inhibition of *hdc1-2* on root growth was due to reduced apical meristem cell proliferation rather than cell elongation. Notably, the root growth of *hdc1-2* showed reduced sensitivity compared to WT after auxin treatment under LK conditions. HDC1 may regulate root growth by affecting auxin polar transport and subsequent auxin signaling, as evidenced by the altered expression of auxin transport genes. Moreover, the organ-specific RT-qPCR analyses unraveled that HDC1 negatively regulates the expression of CBL-CIPK-K^+^ channel-related genes such as *CBL1, CBL2*, *CBL3*, *AKT1,* and *TPK1*, thereby establishing a molecular link between histone deacetylation, auxin signaling, and CBLs-CIPKs pathway in response to K^+^ deficiency.

## Simple Summary

To elucidate the regulatory role of Histone Deacetylase Complex 1 (HDC1) in the primary root growth of *Arabidopsis thaliana* under potassium (K^+^) deficiency, we examined primary root growth changes in the *hdc1-2* mutant under K^+^ deficiency stress. The *hdc1-2* mutant exhibited significantly inhibited primary root growth compared to the wild-type (WT) plants under low-potassium (LK) conditions, indicating that HDC1 positively regulates root growth under LK conditions. We measured various root zones and found that the inhibition of root growth in *hdc1-2* was attributed to reduced apical meristem cell proliferation. The root growth of *hdc1-2* showed reduced sensitivity compared to WT after auxin treatment under LK conditions. Moreover, HDC1 negatively regulated the expression of the CBL-CIPK module genes. These findings suggest that HDC1 connects histone deacetylation, auxin signaling, and the CBLs-CIPKs pathway in response to K^+^ deficiency.

## 1. Introduction

Potassium (K^+^) is the predominant inorganic cation in plant cells and plays a crucial role in fundamental physiological processes and stress responses [1]. It is involved in enzyme activation, protein synthesis, photosynthesis, and the regulation of water uptake and transport within plants. Additionally, K^+^ contributes to the maintenance of turgor pressure in plant cells [2,3]. In most arable fields, the concentration of soluble K^+^ varies between 0.1 and 1 mM and fluctuates greatly with additional potash fertilization, presenting a challenge for plants in maintaining optimal K^+^ levels for growth and development [4,5]. However, plants have evolved the ability to efficiently absorb K^+^ from their surroundings and maintain relatively high levels within the cytosol [6]. In-depth analysis of the molecular mechanisms of plant response and adaptation to limited K^+^ in the soil can guide the breeding of new crops with higher K^+^ utilization efficiency and support sustainable agriculture.

The customary K^+^ concentration in plant cells is generally higher than that observed in the soil of most arable fields [7]. Consequently, most research has focused on how plants respond to low-K^+^ (LK) environments. K^+^ homeostasis is regulated by a diverse array of K^+^ channels and transport proteins, facilitating plant adaptation to K^+^ deficiency [8,9]. Ca^2+^-CBLs-CIPKs pathways play key roles in connecting K^+^ status with the activity of channels and transporters [10]. Specifically, to deal with K^+^ deficiency, the CBL1/9-CIPK1/9/23 complex enhances the uptake of K^+^ ions from the environment by activating the AKT1 K^+^ channel and the HAK5 K^+^ transporter on the plasma membrane through phosphorylation. Concurrently, the CBL2/3-CIPK3/9/23/26 complex can activate the TPK1/3/5 K^+^ channel on the vacuolar membrane, thereby releasing vacuolar K^+^ into the cytoplasm [11,12,13,14]. HAB1/ABC1/ABC2/PP2CA phosphatases in the ABA signaling pathway can dephosphorylate vacuolar membrane CBL2/3 and influence its degradation under high-potassium (HK) conditions, serving a negative regulatory role in the plant response to LK stress [15]. Moreover, the target of the rapamycin complex (TORC), an evolutionarily conserved nutrient regulator in eukaryotes [16,17,18], can negatively regulate the activity of CBL2/3-CIPKs in plants, which in turn can inhibit TORC [10]. However, the understanding of the upstream regulatory networks of the Ca^2+^-CBL-CIPK pathway and TORC module in LK responses remains limited.

Auxin is a vital phytohormone regulating plant growth and development, and it acts on the distribution of plant roots [19]. In the roots of *Arabidopsis thaliana* L., auxin is symmetrically distributed in the root cap and meristem, and the auxin concentration in each tissue is coordinated by inflow and efflux [20]. Auxin efflux carriers formed by PIN-FORMED (PIN) proteins and auxin influx carriers formed by AUXIN RESISTANT 1 (AUX1) protein in the plasma membrane are essential for polar auxin transport [21,22,23,24]. Research has shown an intriguing interplay between the K^+^ and auxin signaling pathways. K^+^ deficiency at the root tip can inhibit auxin transport, leading to uneven auxin distribution in the central cylindrical cells of the apical part of the root tip, which inhibits primary root growth [25,26,27].

In eukaryotes, multiple post-translational modifications of histones in chromatin, such as acetylation, methylation, phosphorylation, and ubiquitination, can dynamically and reversibly regulate the chromatin structure, thereby regulating the expression patterns of genes wrapped around core histones [28,29]. Increasing evidence indicates that histone modification plays essential roles in plant responses to environmental stress. For example, H3K27me and H3K27ac mediate transcriptional dynamics and contribute to root development and nitrogen (N) metabolic processes in wheat [30]. Histone deacetylase 6 (HDA6) physically interacts with ABI5 to decrease apple drought tolerance [31]. HDA19 is involved in both root cell elongation and the expression regulation of some phosphate (P) starvation response genes under inorganic P (Pi)-deficient conditions [32]. Histone Deacetylase Complex 1 (HDC1) is a component of the histone deacetylation complex. A previous study showed that HDC1-mediated histone H3 deacetylation represses the transcriptional activation of genes involved in Pi starvation responses in *A. thaliana* [33]. Knockout/overexpression of HDC1 increases/decreases the sensitivity of plants to abscisic acid (ABA) and salt stress [34]. A recent study revealed that HDC1 responds to salt stress via a dual mechanism involving both histone deacetylation and histone methylation [35]. However, the function and mechanism of HDC1 in response to other stresses, such as low K^+^ and low N, still need to be elucidated.

In this research, we used the HDC1 mutant line, *hdc1-2,* to investigate the role of histone acetylation in the primary root growth response to K^+^ deficiency in *A. thaliana*. HDC1 regulated root growth under LK stress by regulating PIN-mediated polar auxin transport. We found that the inhibition of *hdc1-1* on primary root growth was increased under K^+^ deficiency compared with WT plants. Auxin signal transduction is necessary for root growth and development. In summary, our results suggest that HDC1 is a negative regulator of primary root growth under conditions of K^+^ deficiency.

## 2. Materials and Methods

### 2.1. Plant Materials

All *A. thaliana* stocks were in the Landsberg *erecta* (L*er*) background. *hdc1-2* was segregated from the *ag-11 hdc1-2* double mutant obtained by EMS mutagenesis with *ag-11*. Gene diagrams of *HDC1* showing the locations of the *hdc1-2* mutation and phenotypes of *ag-11* and *hdc1-2* single and double mutants are shown in Figure S1. The *HDC1* full-length genomic sequence was constructed into pGWB616, driven by the *HDC1* native promoter and transformed into L*er* and *hdc1-2* to obtain overexpression (OE) and complementation (COM) lines. The transformation of *A. thaliana* was carried out through the infection of *A. thaliana* inflorescences with *Agrobacterium* strain (GV3101).

### 2.2. Phenotype Analyses and Plant Growth Conditions

Seeds were surface-sterilized with a mixed solution of 75% (*v*/*v*) ethanol, washed with sterilized distilled water, and then incubated in the dark at 4 °C for 7 days. The seeds were then planted on LK (50 μM) or HK (5 mM) media containing 1% (*w*/*v*) sucrose and 0.9% (*w*/*v*) agar and grown at 22 °C under constant illumination at 60 μmol.m^−2^.s^−1^ for 7 days. LK and HK medium were modified from MS (pH 5.8) according to Zhang et al. [36].

For auxin treatment experiments, NAA (1-naphthaleneacetic acid) or IAA (indole-3-acetic acid) was added after the autoclaved medium was cooled to 60 °C. NAA and IAA were ordered from Sigma-Aldrich (Saint Louis, MO, USA). Photographs were taken after 5 to 7 days. Three technical replicates were analyzed for each biological replicate, and the phenotypic assay was repeated at least three times.

For seed harvesting and hybridization, *A. thaliana* plants were cultured in the potting soil mixture (rich soil: vermiculite, ¼ 2:1, *v*/*v*) and kept in a plant growth room at 22 °C in a 16 h-light/8 h-dark cycle with a light intensity of 120 μmol.m^−2^.s^−1^. The relative humidity of the growth chamber remained at 65–75% (*v*/*v*).

### 2.3. Confocal Microscopy and Root Apical Meristem Cell Number Counting

For primary root tip observation, sterilized *A. thaliana* seeds were directly germinated on HK medium and LK medium for 7 days or 5 days, and then the roots were imaged using Canon EOS R6, and the lengths were measured using Digimizer software (Version 3.12.0). Plants were stained using propidium iodide solution (10 mg/mL) for 1 min, followed by a 2 min rinse using deionized water. Roots were then observed using a laser confocal scanning microscope (Zeiss LSM710, Oberkochen, Germany) with the excitation and emission wavelengths set at 536 nm and 617 nm, respectively. The meristem cortex cell number was counted based on the files of cortex cells extending from the quiescent center to the transition zone, represented by the first elongated cell, which underwent cell expansion along the longitudinal axis with an elongated morphology [37]. Three biological replicates were prepared and analyzed simultaneously.

### 2.4. Histochemical GUS Staining

Then, 1158 bp was selected as the promoter of HDC1 and cloned into pGWB162 with the GUS coding region. The *ProHDC1:GUS* construct was transformed into L*er* using the floral dip method. Three independent transgenic lines expressing *ProHDC1:GUS* were analyzed. After 7 days of growth in HK medium or LK medium, tissues or hand-cut roots of the transgenic plants were incubated in commercial GUS staining solution at 37 °C for about 12 h, and then destained with 75% (*v*/*v*) ethanol. Images were taken under a stereomicroscope, and six roots were analyzed.

### 2.5. RT-qPCR Analysis

Total RNA was isolated from the roots of 7-day-old seedlings using TRIzol reagent (Invitrogen, Waltham, MA, USA). The RNA was subsequently treated with DNase I (RNase Free, Takara, Kusatsu, Japan) to eliminate any potential genomic DNA contamination. One microgram of total RNA was reverse-transcribed using NovoScript Plus All-in-one 1st Strand cDNA Synthesis Super Mix (Novoprotein). Quantitative real-time PCR analysis was performed on a Bio-Rad CFX-96 Real-time PCR system (Bio-Rad, Hercules, CA, USA) using 2 × ChamQ Universal SYBR qPCR Master Mix (Vazyme, Nanjing, China). Each sample was subjected to three biological replicates. *ACTIN2* was used as an internal control, and the relative expression levels were calculated using the 2^−ΔΔCT^ method. Three technical replicates were analyzed for each biological replicate. The qPCR primers used are shown in Supplemental Table S1.

### 2.6. Accession Numbers

Sequence data from this article can be found in TAIR (www.Arabidopsis.org) under the following accession numbers: *HDC1* (At5g08450), *PIN1* (At1G73590), *PIN2* (At5G08720), *PIN3* (At1G70940), *AUX1* (At2G38120), *CBL1* (At4g17625), *CBL9* (At5g47100), *CBL2* (At5G55990), *CBL3* (At4G26570), *AKT1* (At2G26650), *TPK1* (At5G55630).

## 3. Results

### 3.1. Root Apical Meristem Activity Is Reduced in hdc1-2 Mutant Under Low-K^+^ Conditions

**Figure 1:**
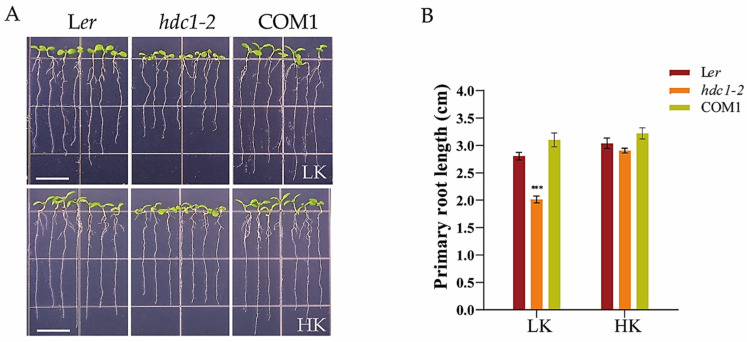
*Arabidopsis thaliana* L. primary root growth in *hdc1-2* was inhibited under low-potassium (LK) conditions. (**A**) Phenotypes of the wild-type (L*er*), *hdc1-2* mutant (*hdc1-2*), and *hdc1-2*/*ProHDC1:HDC1* complementation line (COM1). Arabidopsis seeds were germinated and grown on LK (50 μM) or HK (5 mM) media. The phenotypes were recorded on the seventh day. (**B**) Primary root lengths of the plants tested in (**A**). Data are means of three independent biological replicates ± SE; 21 seedlings per genotype under each condition were processed for each replicate. Statistical significance was determined using Student’s *t*-test (*** *p* < 0.001), Scale bar, 1 cm.

To investigate the role of HDC1 in root growth under low-K^+^ stress, we observed the growth of a homozygous mutant *hdc1-2* (Figure S1), which was generated through EMS mutagenesis in the L*er* ecotype of *A. thaliana* and verified through complementary transformation experiments under various K^+^ conditions. When Arabidopsis seeds were germinated and cultured on low-K^+^ (LK, 50 μM) medium for 7 days, the *hdc1-2* mutant exhibited significantly greater inhibition of primary root growth than L*er* and the complementation line (COM1) plants (Figure 1). However, in the high-K^+^ (HK, 5 mM) medium, *hdc1-2* showed a marginally shorter primary root than L*er* and COM1 (Figure 1). These observations suggest that the short root phenotype of the *hdc1-2* mutant under LK stress was attributable to the loss of *HDC1* function.

**Figure 2:**
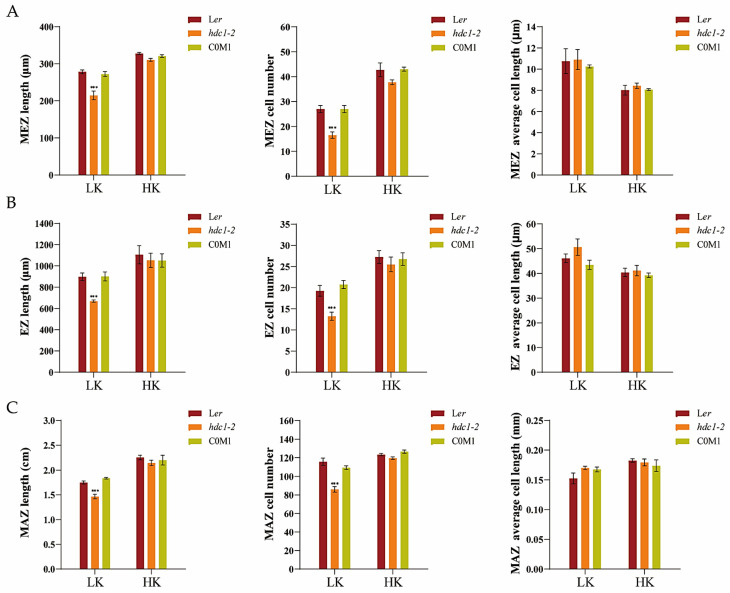
The short-root phenotype of the *hdc1-2* mutant under low-potassium (K^+^) conditions is attributed to the suppression of root apical meristem cell proliferation. (**A**–**C**) Different regional lengths, regional cell numbers, and cell lengths of Arabidopsis roots. Seeds were germinated and grown in low-potassium (LK, 50 μM) or high-potassium (HK, 5 mM) medium. The phenotypes were recorded on the seventh day; MEZ, apical meristem zone; EZ, elongation zone; MAZ, maturation zone. Data are means of three independent biological replicates ± SE, and four seedlings per genotype under each condition were processed for each replicate. Statistical significance was determined using Student’s *t*-test (*** *p* < 0.001).

Efficient cell division and differentiation in a dynamic balance are key to maintaining continuous root growth [38,39], and root developmental plasticity relies on changes in apical meristem activity [40]. To elucidate the underlying mechanism responsible for the short primary roots of *hdc1-2*, we observed and measured the different zones within the primary roots, specifically the apical meristem zone (MEZ), elongation zone (EZ), and the maturation zone (MAZ). No significant differences in root growth were observed between L*er* and *hdc1-2* under HK conditions. However, under LK stress, all the tested zones were shorter in *hdc1-2* than in L*er* due to a decrease in the number of cells in the mutant root rather than cell length (Figure S2 and Figure 2). This suggests that under LK conditions, the cell division of *hdc1-2* may have been impaired, which led to a decrease in the number of root cells.

### 3.2. Overexpression of the HDC1 Gene Enhances Root Apical Meristem Under Low-K^+^ Conditions

**Figure 3:**
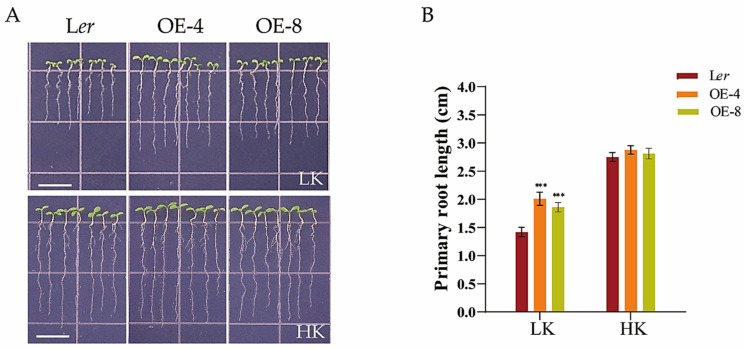
The *Arabidopsis thaliana* L. primary root growth in the HDC1-overexpression line was promoted under low-potassium (K^+^) conditions. (**A**) Phenotypes of the wild-type (L*er*) and L*er*/*ProHDC1:HDC1* overexpression lines (OE-4 and OE-8). Seeds were germinated and grown on low-potassium (LK, 50 μM) or high-potassium (HK, 5 mM) medium. The phenotypes were recorded on the fifth day. Scale bar, 1 cm. (**B**) Primary root lengths of plants tested in (**A**). Data are means of three independent biological replicates ± SE, and 21 seedlings per genotype under each condition were processed for each replicate. Statistical significance was determined using Student’s *t*-test (*** *p* < 0.001).

To further corroborate the above conclusion, we generated overexpression lines of *ProHDC1:HDC1*. After 5 days of growth on LK medium, the primary root length of these two overexpression lines (OE-4 and OE-8) was significantly longer than that of the wild-type (L*er*) (Figure 3). This phenotype indicates that *HDC1* plays a role in regulating root growth under LK conditions.

**Figure 4:**
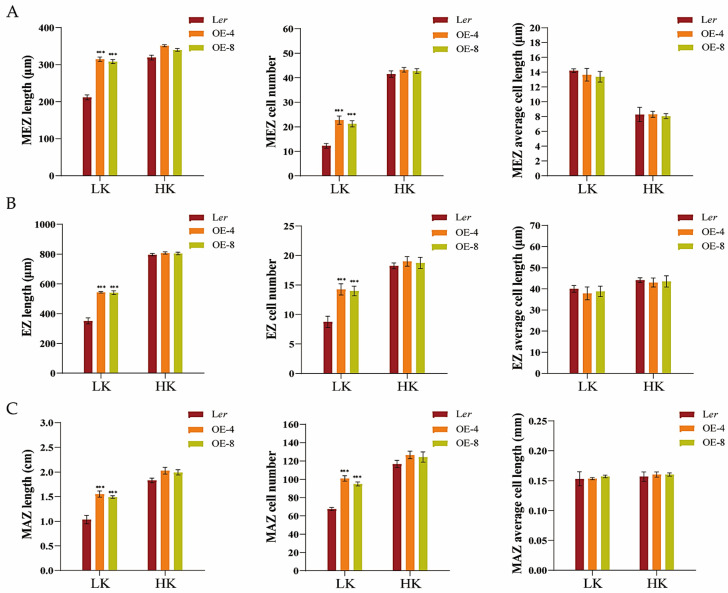
The long-root phenotype of *HDC1* overexpression lines under low-potassium (K^+^) conditions is attributed to the enhancement of root apical meristem cell proliferation. (**A**–**C**) Different regional lengths, regional cell numbers, and cell lengths of young roots of Arabidopsis. Seeds were germinated and grown on low-potassium (LK, 50 μM) or high-potassium (HK, 5 mM) medium. The phenotypes were recorded on the fifth day. MEZ, apical meristem zone; EZ, elongation zone; MAZ, maturation zone. Data are means of three independent biological replicates ± SE, four seedlings per genotype under each condition were processed for each replicate. Statistical significance was determined using Student’s *t*-test (*** *p* < 0.001).

At the same time, observations and measurements were conducted on different regions of the main root of overexpressed (OE) lines. Under LK conditions, due to an increase in the number of cells in the root, all test regions of the OE-4 and OE-8 transgenic plants were longer than those of L*er*, but there was no difference under HK conditions (Figure S3 and Figure 4). These results further indicated the regulation of root mitotic cell division by HDC1.

### 3.3. Expression Pattern of HDC1 and Its Responses to K^+^ Availability

**Figure 5:**
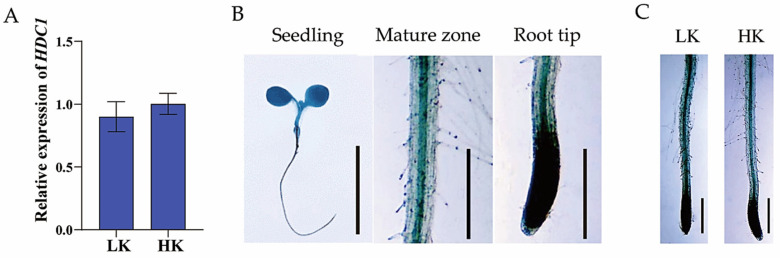
Expression patterns of *HDC1* in response to potassium (K^+^) availability in *Arabidopsis thaliana* L. (**A**) Quantitative reverse transcription polymerase chain reaction (RT-qPCR) analysis of *HDC1* expression in roots in response to K^+^ availability. The expression levels were normalized to those of *AtACT2,* an internal control. Data are means ± SE of three independent biological replicates; each with three technical replicates; each sample contained 150–200 individual plants. (**B**) β-Glucuronidase (GUS) activity assay of *HDC1* expression. Bars, 1 cm (seedling); 1 mm (mature zone and root tip). (**C**) GUS staining in the root tips of *ProHDC1: GUS* transgenic plants. Scale bar, 1 mm. Seeds were germinated and grown on low-potassium (LK, 50 μM) or high-potassium (HK, 5 mM) medium for 7 days. Ten seedlings were stained, and six roots were analyzed.

The phenotypes of impaired root growth and cell division suggest a potential role for HDC1 in root meristem responses to K^+^ deficiency. Consequently, we examined the transcript abundance of *HDC1* in roots under LK and HK conditions. As shown in Figure 5A, the expression level of *HDC1* remained unaffected by K^+^ deficiency compared with that in plants grown under sufficient K^+^. To verify and further analyze the HDC1 expression pattern and its response to K^+^ availability, we generated transgenic plants (*ProHDC1:GUS*) carrying the marker gene GUS driven by the native *HDC1* promoter in the L*er* ecotype. The results of GUS staining showed that HDC1 was widely expressed in the vegetative seedling stage of Arabidopsis, with GUS activity more pronounced in the root tip than in other parts of the root (Figure 5B), which is consistent with a previous study in the Col-0 ecotype [33] and a potential role of HDC1 in root meristem responses to K^+^ deficiency. Subsequently, randomly selected *ProHDC1:GUS* lines were subjected to LK or HK treatment for 7 days. The samples were then collected for staining observation. As shown in Figure 4C, strong GUS activity at the root tips could not be differentiated between the LK and HK treatments.

### 3.4. The Root Growth Phenotype of hdc1-2 Under Low-K^+^ Conditions Is Controlled by Auxin Signaling

**Figure 6:**
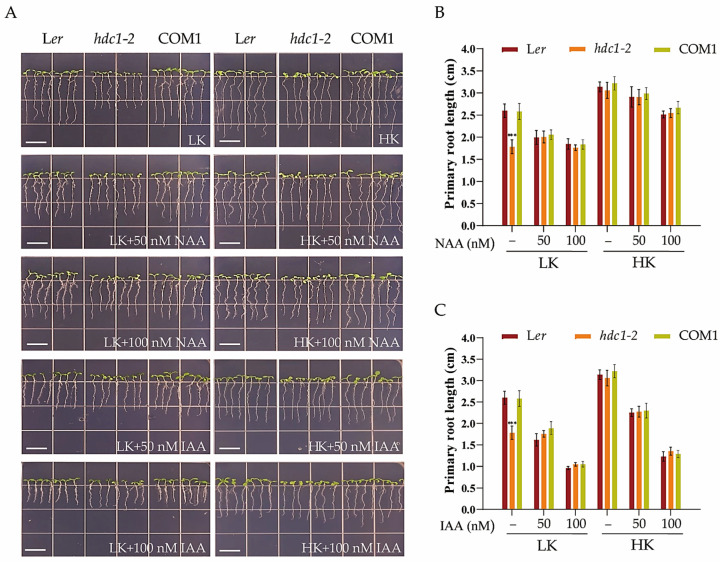
Partial restoration of *Arabidopsis thaliana* L. primary root growth in the *hdc1-2* mutant with low-potassium (K^+^) inhibition by exogenous application of NAA and IAA. (**A**) Phenotypes of the various plants. Scale bar, 1 cm. (**B**,**C**) Primary root length of the plants tested in (**A**). Seeds were germinated and grown on low-potassium (LK, 50 μM) or high-potassium (HK, 5 mM) medium with or without NAA or IAA. The phenotypes were recorded on the seventh day. Data are means of three independent biological replicates ± SE, and 21 seedlings per genotype under each condition were processed for each replicate. Statistical significance was determined using Student’s *t*-test (*** *p* < 0.001).

**Figure 7:**
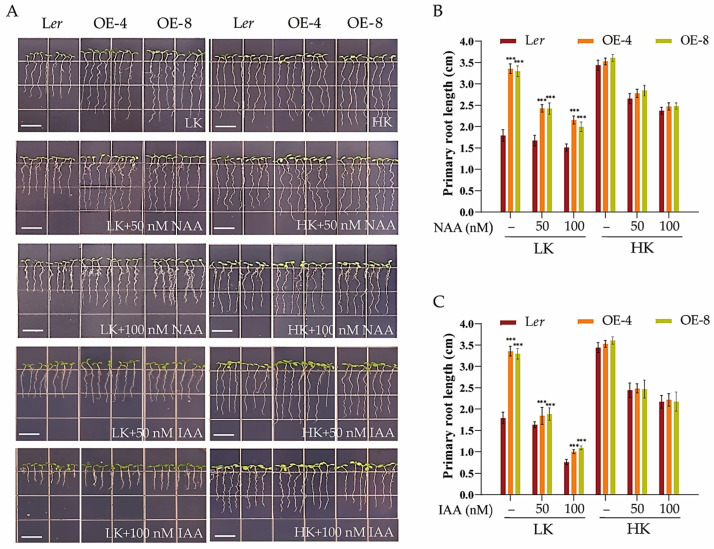
Inhibition of *Arabidopsis thaliana* L. primary root growth in overexpression lines by exogenous application of NAA and IAA under low-potassium (K^+^) stress. (**A**) Phenotypes of various plants. Scale bar, 1 cm. (**B**,**C**) Primary root length of the plants tested in (**A**). *Arabidopsis* seeds were germinated and grown on low-potassium (LK, 50 μM) or high-potassium (HK, 5 mM) medium with or without NAA or IAA. The phenotypes were recorded on the fifth day. Data are means of three independent biological replicates ± SE, and 21 seedlings per genotype under each condition were processed for each replicate. Statistical significance was determined using Student’s *t*-test (*** *p* < 0.001).

Previous research has uncovered the crucial role of auxin in regulating cell division, elongation, and differentiation, as well as in the response of roots to environmental stimuli [41,42]. Specifically, studies have demonstrated that the root growth phenotype under low-K^+^ conditions is regulated by auxin signaling in Arabidopsis [43]. L*er* and *hdc1-2* plants exhibited different root growth phenotypes under LK conditions. We hypothesized that auxin signaling might be involved in this root growth phenotype. To investigate whether the short-root phenotype of *hdc1-2* was due to a decrease in auxin levels, we supplemented the medium with 1-naphthalene acetic acid (NAA), and the root growth phenotype was indeed affected. In the LK medium, the addition of NAA inhibited the primary root growth of L*er* and COM1, and no significant difference in primary root length was observed among L*er*, COM1, and *hdc1-2*, as the root growth of *hdc1-2* remained largely unaltered after NAA treatment (Figure 6). This observation suggests that the sensitivity of *hdc1-2* to NAA/IAA was diminished under LK conditions. Correspondingly, the overexpression lines (OE-4 and OE-8) maintained a longer primary root length than L*er*, despite the inhibitory effect of NAA addition to the LK medium. Similar phenotypes were observed under the exogenous IAA treatment (Figure 7). These findings suggest that HDC1 positively regulates the sensitivity of *Arabidopsis* primary roots to auxin under LK conditions.

**Figure 8:**
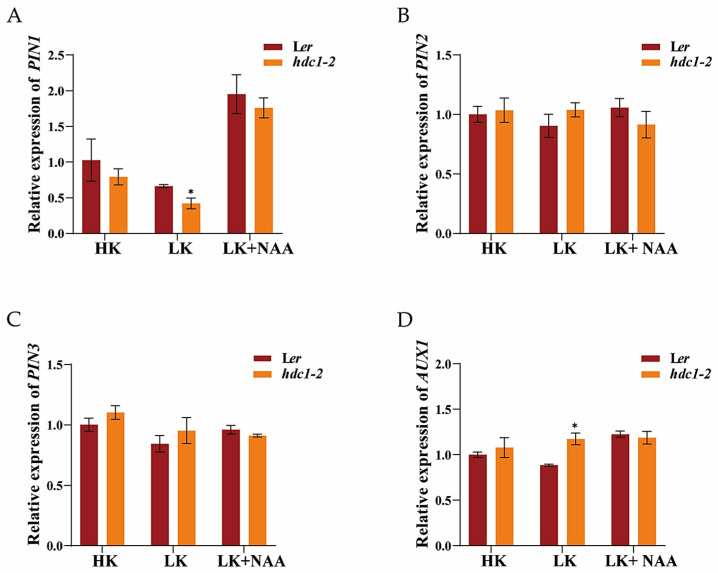
Expression of *PIN1* (**A**), *PIN2* (**B**), *PIN3* (**C**)*,* and *AUX1* (**D**) under low-potassium (LK, 50 μM), high-potassium (HK, 5 mM), and LK + 1-naphthaleneacetic acid (NAA, 100 nM) treatments. *Arabidopsis* seeds were germinated and grown in different media for 7 days. Expression levels were normalized to *AtACT2,* an internal control. Data are presented as means ± SE of three independent biological replicates; each with three technical replicates; each sample contained 150–200 individual plants. Statistical significance was determined using Student’s *t*-test (*, *p* < 0.05).

Auxin export vector proteins PIN1, PIN2, PIN3, and the input vector protein AUX1 mediate rapid auxin signaling in roots [44,45,46]. To further investigate whether the auxin transport pathway is involved in the growth retardation phenotype of *hdc1-2* under LK conditions, we examined the expression of *PIN1*, *PIN2*, *PIN3*, and *AUX1*. The RT-qPCR results indicate that the expression of *PIN1* was upregulated and the expression of *AUX1* was significantly downregulated in *hdc1-2* under LK conditions (Figure 8). Upon application of exogenous NAA, a significant increase in the expression of *PIN1* in *hdc1-2* was observed, suggesting that exogenous NAA can partially restore the inhibited auxin transport levels in the LK treatment (Figure 8A). Considering the reduced sensitivity of *hdc1-2* to NAA/IAA under LK conditions, it can be inferred that HDC1 regulates root growth by influencing PIN1- and AUX1-mediated auxin transport and subsequently auxin signaling.

### 3.5. HDC1 Negatively Regulates the Expression of CBL-CIPK Module Genes

**Figure 9:**
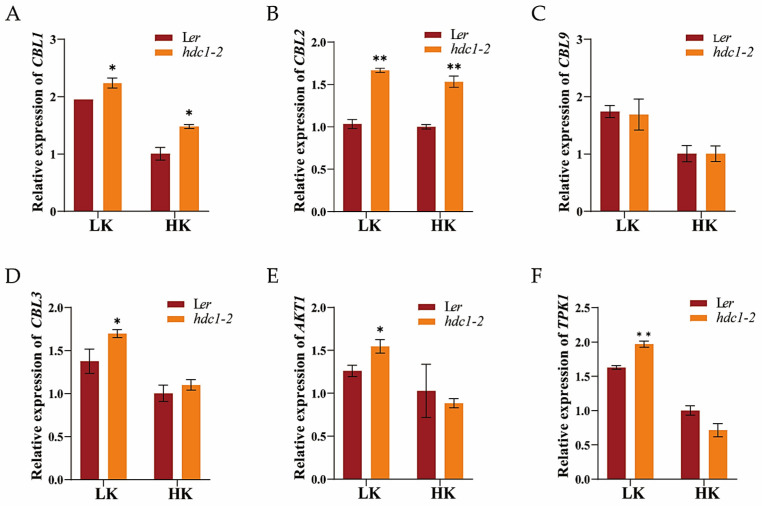
*HDC1* affects the expression of “CBL-CIPK-channel” module genes in *Arabidopsis thaliana* L. (**A**–**F**) Transcript levels of *CBL1*, *CBL2, CBL9*, *CBL3, AKT1,* and *TPK1* in WT (L*er*) and *hdc1-2* mutant under low-potassium (LK, 50 μM) and high-potassium (HK, 5 mM) conditions. *Arabidopsis* seeds were germinated and grown in LK or HK medium for 7 days. Expression levels were normalized to *AtACT2,* an internal control. Data are presented as means ± SE of three independent biological replicates; each with three technical replicates; each sample contained 150–200 individual plants). Statistical significance was determined using Student’s *t*-test (*, *p* < 0.05; **, *p* < 0.01).

It is well established that dual CBL-CIPK pathways regulate K^+^ channels and transporters in response to K^+^ fluctuations in the environment. HDC1 has been identified as a component of HDAC complexes, and its knockout promotes histone acetylation and gene expression [34]. We examined the transcript levels of genes in “CBL-CIPK-channel” modules in response to external K^+^ and found that their expression was induced by LK (Figure 9), consistent with previous reports. There were no differences in the transcript levels of *CBL9* between *hdc1-2* and L*er*, although the expression of *CBL9* was induced by LK stress, similarly to other genes (Figure 9C). However, the expressions of *CBL1* and *CBL2* were considerably higher in *hdc1-2* than in L*er* under both the HK and LK conditions (Figure 9A,B). Additionally, the expressions of *CBL3*, *AKT1*, and *TPK1* were significantly increased in *hdc1-2* under LK stress (Figure 9D–F). These results indicate that HDC1 acts upstream of “CBL-CIPK-channel” modules under K^+^ deficiency.

## 4. Discussion

Histone acetylation/deacetylation, mediated by histone acetyltransferases (HATs) and histone deacetylases (HDACs), is a reversible epigenetic switch that regulates plant gene expression. Studies have demonstrated that HDC1 is a shared subunit of HDA6 and HDA19 HDAC complexes [47]. HDC1 enables multiple protein interactions in HDAC complexes and participates in the regulation of flowering time, fruit growth, abscisic acid sensitivity, and responses to salt and low-P stress [33,34,35]. Plant roots perceive abiotic stress in the soil and adapt their architecture to maintain survival and development [48,49]. To better characterize the involvement of HDC1 in the nutritional stress response of plants, we investigated the effect of K^+^ supplementation on primary root growth in Arabidopsis mutant and overexpression lines of *HDC1*. Our findings indicate that *HDC1* is essential for the maintenance of primary root growth under LK conditions in Arabidopsis (Figure 1 and Figure 3). Further analysis demonstrated that *HDC1* is crucial for the preservation of apical meristem cell division under LK conditions (Figure 2 and Figure 4), thereby exerting a regulatory function on primary root growth. This is supported by the observation that HDC1 exhibits strong expression in root tips (Figure 5). A previous study has shown that HDC1 is post-translationally regulated in response to Pi deficiency. Our in planta promoter-GUS activity assay results reveal that strong GUS activity at the root tips could not be differentiated between LK and HK treatments, consistent with the root-specific RT-qPCR analysis results of *HDC1* (Figure 5). Considering these findings collectively, we propose that HDC1 is post-translationally regulated in response to K^+^ deficiency.

The plasticity of the plant root system is largely related to phytohormone metabolism and signaling. For example, higher CuSO4 concentrations inhibited primary root elongation in Arabidopsis seedlings by modulating auxin distribution through PIN1 but not PIN2 or AUX1 [50,51]. Previous research has indicated a link between LK perception and auxin signaling [43]. Later research showed that the AKT1-mediated LK response is primarily mediated by PIN1 proteins [52]. In the current study, we found that HDC1 positively regulates the sensitivity of root growth to auxin because the root growth of *hdc1-2* was almost unchanged, and *HDC1*-overexpression lines still had longer primary roots than L*er* after NAA treatment under LK (Figure 6 and Figure 7). HDC1 may regulate root growth both by affecting auxin transport and subsequently auxin sensitivity, since the expression of *PIN1* and *AUX1* was significantly altered (Figure 8).

Plants mainly rely on two strategies to cope with low potassium stress in the environment: the CBL1/9-CIPKs complex and the CBL2/3-CIPKs complex. The CBL1/9-CIPK1/9/23 complex activates the K^+^ channel AKT1 and K^+^ transporter HAK5 in the plasma membrane in response to low-K^+^ stress. Tonoplast-localized CBL2 and CBL3 interact with four CIPKs that, in turn, propel K^+^ remobilization from the vacuole store through the activation of TPK-type K^+^ channels [53,54]. Previous research has demonstrated that “CBL-CIPK-channel” modules respond to external K^+^ by altering their protein abundance [15]. However, limited information is available regarding the upstream regulators of “CBL-CIPK-channel” modules in plants. Our organ-specific RT-qPCR analyses revealed that HDC1 negatively regulates the expression of *CBL1, CBL2*, *CBL3*, *AKT1*, and *TPK1* (Figure 9). This observation aligns with the role of HDC1-mediated histone deacetylation in the regulation of chromatin structure and gene expression. A recent study reported that HDC1 attenuates salt stress responses by moderating the salt-induced H3K9/14 hyperacetylation and transcriptional activation of stress-induced genes [35]. Considering that “CBL-CIPK-channel” modules were induced by LK, it is plausible that HDC1 also attenuates LK responses via the deacetylation of LK-inducible CBL-CIPK-K^+^ channel-related genes.

In addition to catalyzing histone deacetylation, some HDACs deacetylate non-histone proteins to regulate multiple pathways [55]. HDC1 in plants is encoded by a single copy gene and functions as a component of HDACs. Previous studies have shown that HDC1 mediates histone H3 deacetylation to inhibit *LPR* and *ALMT1* transcription and to regulate root system development in Arabidopsis under Pi starvation [33]. The present results indicate that HDC1 regulates the auxin-dependent primary root growth and the expression of CBLs–CIPKs pathway genes. The CBLs-CIPKs pathway regulates auxin transporters and coordinates auxin-mediated root responses to abiotic stresses [56,57]. We hypothesized that HDC1 mediates histone H3 deacetylation to inhibit CBLs-CIPKs signaling pathways, thus modulating auxin transporters and regulating auxin-mediated root responses to LK stress.

## 5. Conclusions

**Figure 10:**
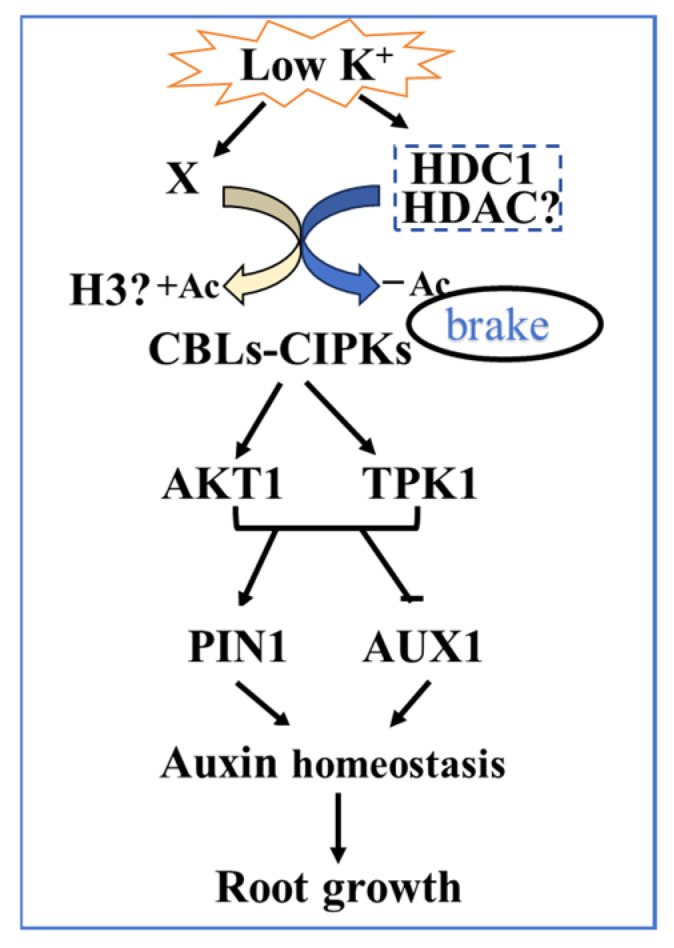
The working model of *HDC1* in root growth regulation in response to LK stress. HDC1 puts the brake on low-potassium (K^+^) responses as it stabilizes the HDAC complex and the mediates histone deacetylation of CBL-CIPK-K^+^ channel-related genes, thereby counteracting low-K^+^-stress-induced acetylation and dampening transcriptional upregulation, thus modulating auxin transporters and regulating auxin-mediated root responses to low K^+^ stress.

In this study, our findings establish a novel molecular pathway of HDC1-controlled root growth and highlight the molecular link between the CBLs-CIPKs pathway and auxin signaling in response to K^+^ deficiency. We propose that HDC1 puts the brake on LK responses via the deacetylation of LK-inducible “CBL-CIPK-K^+^ channel” modules to counteract LK-induced histone hyperacetylation (Figure 10). This mechanism reinforces the role of HDC1 as a multifunctional scaffolding protein that interacts with HDACs. Future research should investigate the histone acetylation levels of the CBLs–CIPKs pathway and auxin pathway gene sites and identify the HDC1 interactor in response to LK stress.

## Data Availability

The data presented in this study are available upon request from the corresponding author.

## Supplementary Materials

The following supporting information can be downloaded at: https://www.mdpi.com/article/10.3390/biology14010057/s1, Figure S1: Diagram of the *Histone Deacetylase Complex 1* (*HDC1*) gene and phenotypes of *ag-11* and *hdc1-2* single and double mutants. Figure S2: Micrograph of root apical meristem zones of the wild-type (L*er*), *hdc1-2* mutant (*hdc1-2*), and *c16s/ProHDC1:HDC1* complementation line (COM1). Figure S3: Micrograph of root apical meristem zones of wild-type (L*er*) and L*er*/*ProHDC1:HDC1* overexpression lines (OE-4 and OE-8). Table S1: Primers used in this study.
