# Supplementary material for: HDC1 Promotes Primary Root Elongation by Regulating Auxin and K+ Homeostasis in Response to Low-K+ Stress
Source: Biology (Basel). 2025 Jan 12;14(1):57. doi: 10.3390/biology14010057 (PMC11762372; doi:10.3390/biology14010057)
Supplement: Supplementary file 1 [file biology-14-00057-s001.zip › biology-3386208-supplementary.pdf]

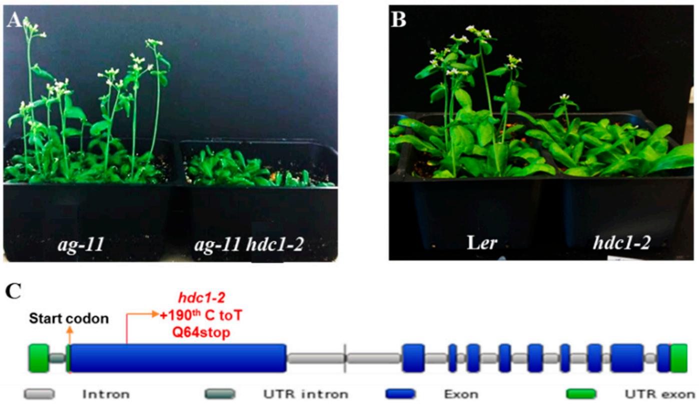

Figure S1. Diagram of the *Histone Deacetylase Complex 1* (*HDC1*) gene and phenotypes of *ag-11* and *hdc1-2* single and double mutants. (A, B) Plants of the indicated genotypes. The plants from *ag-11 hdc1-2* and *hdc1-2* exhibited late-flowering phenotypes. (C) Gene diagram of HDC1 showing the locations of the hdc1-2 mutation.

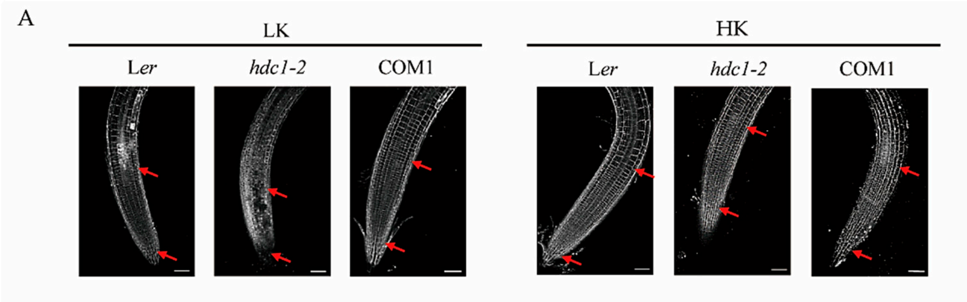

Figure S2. Micrograph of root apical meristem zones of wild-type (L*er*), *hdc1-2* mutant (*hdc1-2*), and the *c16s/ProHDC1:HDC1* complementation line (COM1). The apical meristem zone are marked with red arrows. Scale bars, 50 μm.

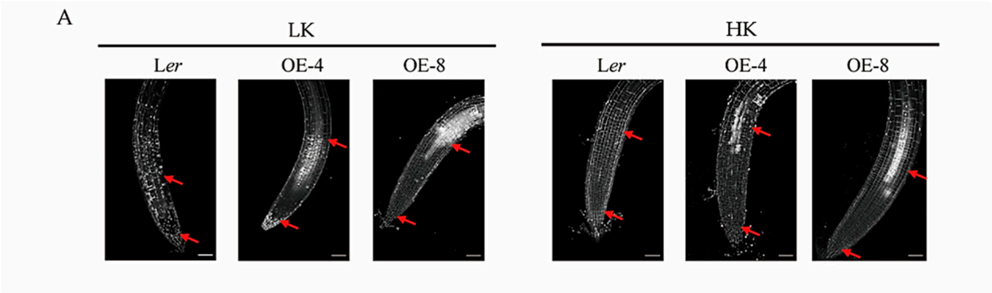

Figure S3. Micrograph of root apical meristem zones of wild-type (L*er*), L*er*/*ProHDC1:HDC1* overexpression lines (OE-4 and OE-8). The apical meristem zone lengths are marked with red arrows. Scale bars, 50 μm.

Table S1. Primers used in this study.

**Primers for quantitative RT-PCR (qRT-PCR)**	
**Primer Name**	**Sequence (5′ to 3′)**
qActin2-F	GGAAGGATCTGTACGGTAAC
qActin2-R	GGACCTGCCTCATCATAC
qHDC1-F	GGGTGCGTCTCTCTGTCTTC
qHDC1-R	ATGATACATTACAAGCCAGGCCA
qPIN1-F	CCTCCGTTTCCTCGCCGCAG
qPIN1-R	TTAGCTCCACGGTACTCAAA
qPIN2-F	CCTCGCCGCACTCTTTCTTTGG
qPIN2-R	CCGTACATCGCCCTAAGCAATGG
qPIN3-F	GAGATCCGTATGTTAGTCCC
qPIN3-R	GGCGTCTTTTGGTCTCTCTG
qAUX1-F	GACGCACTTCTCGACCACTC
qAUX1-R	CCCAATCACTTTCTCCCACA
qCBL2-F	GCTCGTGCTCTCTCCGTCTTTC
qCBL2-R	GCCGCTGCTTGCTTTTGCTTTTG
qCBL3-F	CTGAGTCCGGCATGAACCTGTC
qCBL3-R	TTCCCAAATTGTCTCCTCTGCTAA
qCBL1-F	CGACATGGACTGCACGGGTTAC
qCBL1-R	TCGTGGCAATCTACTCGGTCTTA
qCBL9-F	AGCGCCAAGAGGTGAAGCAG
qCBL9-R	TCTCTTTCACGTCGCAATCTG
qAKT1-F	TGACGAATGTTCTGCTGGAG
qAKT1-R	TGCCATTGTTATCCGATTCA
qTPK1-F	CCGGTTATCTCAGCTTTCTACT
qTPK1-R	AAACTGAGCCAAACATATGCTG
